# Supplementary material for: The Potential Role of Human NME1 in Neuronal Differentiation of Porcine Mesenchymal Stem Cells: Application of NB-hNME1 as a Human NME1 Suppressor
Source: Int J Mol Sci. 2021 Nov 11;22(22):12194. doi: 10.3390/ijms222212194 (PMC8619003; doi:10.3390/ijms222212194)
Supplement: Supplementary file 1 [file ijms-22-12194-s001.zip › ijms-1433365-supplementary.pdf]

International Journal of Molecular Sciences

MDPI

Article

The Potential Role of Human NME1 in Neuronal Differentiation of Porcine Mesenchymal Stem Cells: Application of NB-hNME1 as a Human NME1 Suppressor

Jin Hyoung Cho [1,4,†], Won Seok Ju [1,5,†], Sang Young Seo [1], Bo Hyun Kim [2], Ji-Su Kim [3], Jong-Geol Kim [1], Soon Ju Park [1] and Young-Kug Choo [1,5,*]

Supplementary Information

*****The supplementary information includes following information*****

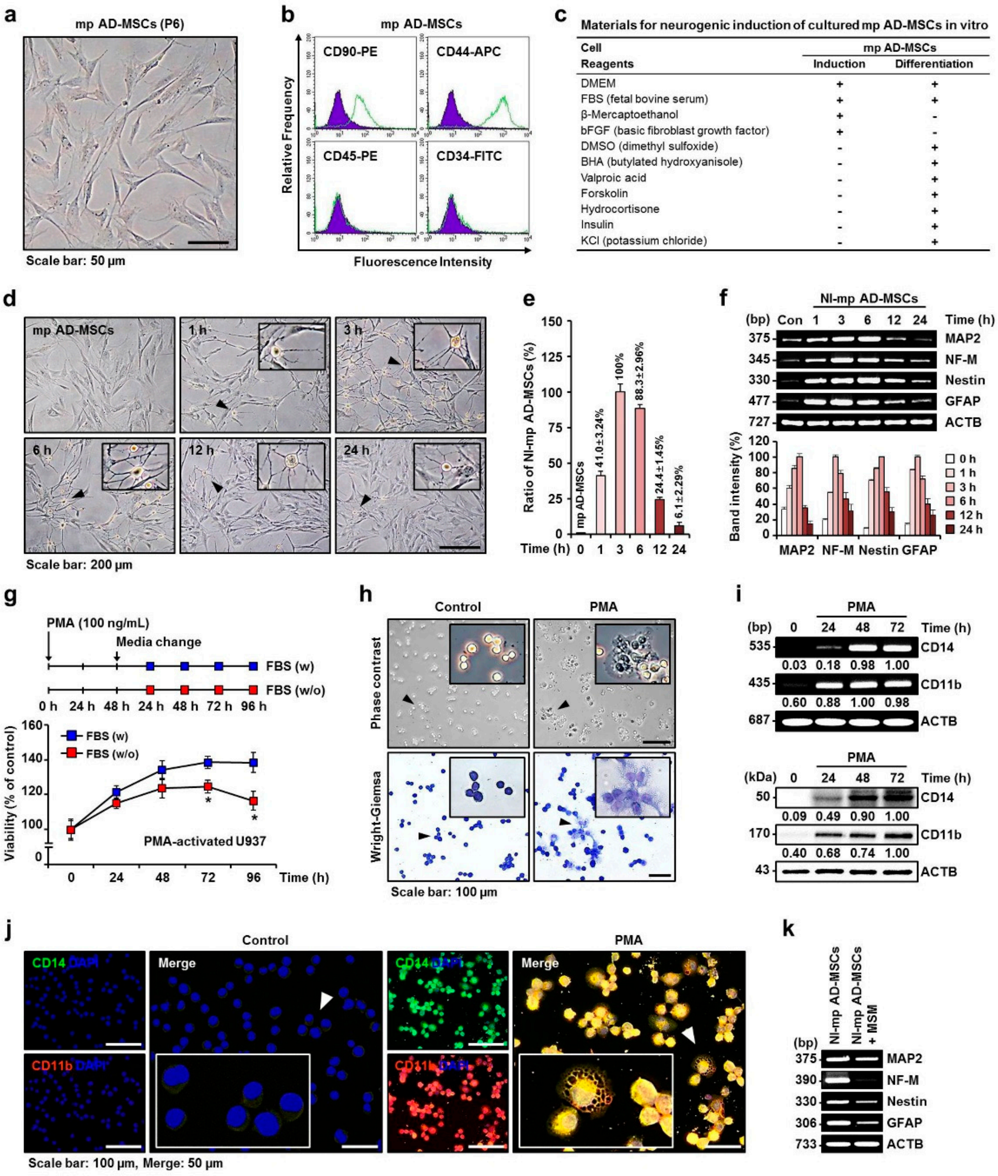

Figure S1. miniature pig adipose tissue-derived mesenchymal stem cells (mp AD-MSCs) and U937 cells. **(a)** Phase-contrast images of mp AD-MSCs in culture (P6, passage 6). Only cells with less than 6 passages were used in this experiment. **(b)** Flow cytometry for surface markers of porcine MSCs. Cell surface analysis of mp AD-MSCs by fluorescence-activated cell sorting (FACS) revealed that mp AD-MSCs were positive for CD90 and CD44 but negative for CD45 and CD34. Positive values are > 90%; negatives are < 5%. Purple indicates the immunoglobulin isotype control; green indicates specific antibody staining profiles. **(c)** Components of cell culture media for both neuronal induction and differentiation of mp AD-MSCs in vitro. **(d)** Time-dependent morphological representation of mp AD-MSC differentiation at 1, 3, 6, 12, and 24 h. Arrowheads indicate enlarged images. **(e)** Variations in the cell count of NI-mp AD-MSCs were quantified at 1, 3, 6, 12, and 24 h. Data are presented as mean percentage levels ± SD (n = 3). **(f)** RT-PCR results confirmed the expression patterns of MAP2, NF-M, nestin, and GFAP in NI-mp AD-MSCs. β-Actin (ACTB) was used as a loading control (upper panel). Quantification of relative band intensity (lower panel) and data are presented as mean percentage levels ± SD (n = 3). **(g)** The phorbol 12-myristate 13-acetate (PMA)-activated macrophages (MPs) were cultured in serum-free or serum-containing media, and cell proliferation was quantified by an MTS assay at 24, 48, 72, and 96 h. MPs and MSM were used within 48 h with no significant reduction in cell growth rates. Data are presented as mean percentage levels ± SD (n = 3; $^*P < 0.05$). **(h)** Phase-contrast images and Wright-Giemsa staining of monocytes and PMA-activated MPs. Arrowheads indicate enlarged images. **(i)** RT-PCR (upper panel) and western blot (lower panel) results confirmed the expression patterns of the CD14 and CD11b cluster in time-dependent manner. ACTB was used as a loading control. **(j)** Immunocytochemistry results showed expression patterns of CD14 and CD11b in monocytes or PMA-activated MPs. Arrowheads indicate enlarged merged images. **(k)** RT-PCR results confirmed the expression patterns of MAP2, NF-M, nestin, and GFAP in NI-mp AD-MSCs or co-cultures with MSM. ACTB was used as a loading control.

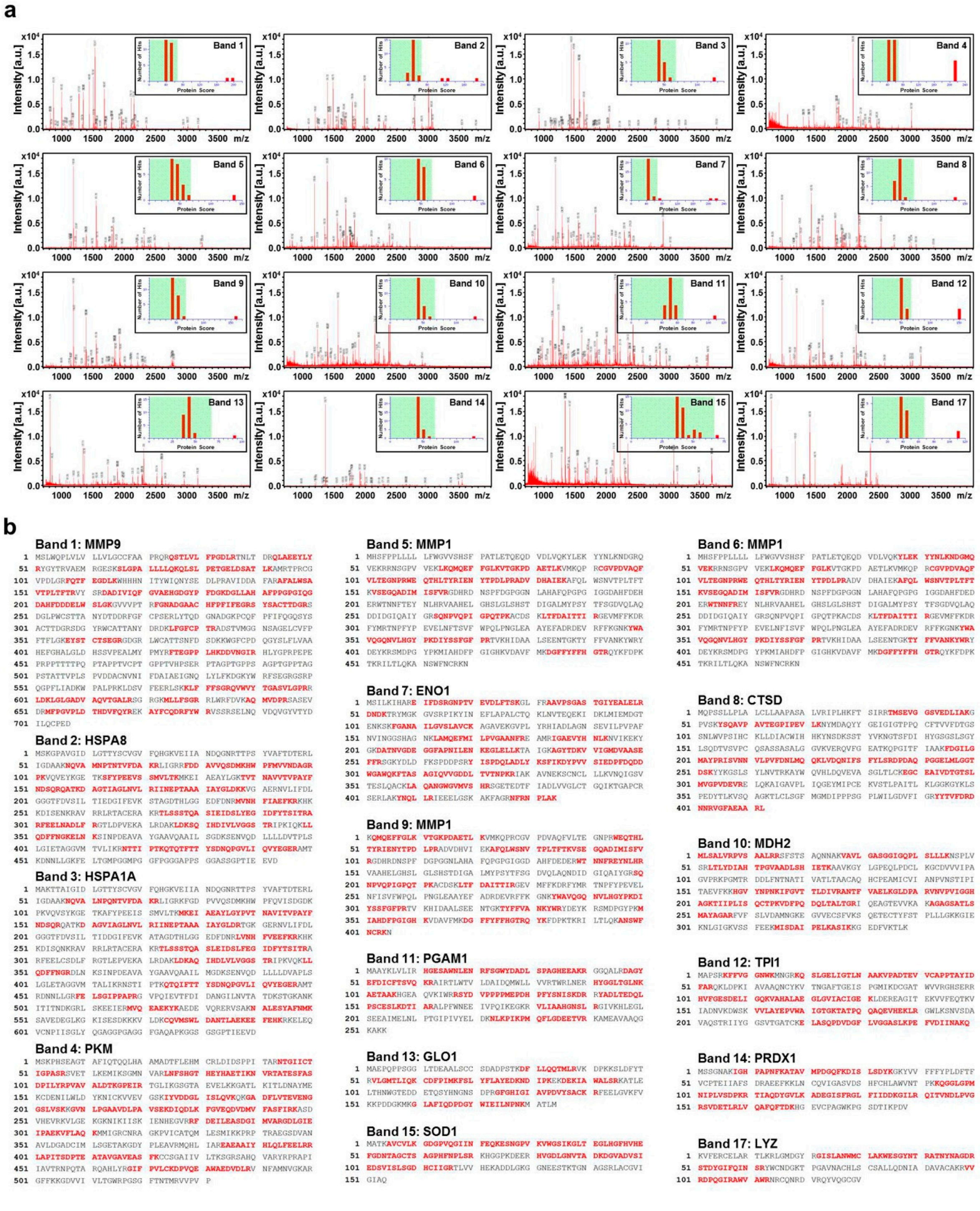

Figure S2. PMF spectrum of MSM containing 17 proteins identified by NCBI Protein database searches using peptide sequences from MS/MS. **(a)** Spectral mass (in mass per charge unit, m/z) obtained by MALDI-TOF MS were analyzed by bioinformatics using the Mascot and Profound search engines. The x-axis represents the mass-to-charge ratio (m/z), and the y-axis represents the relative abundance. **(b)** The probability-based Mowse scores were obtained using the Mascot search engine. Among predicted proteins with differential Mowse scores shown as multiple bars on the x-axis, only those with significant Mowse scores are shown ($P < 0.05$). Red marks indicate peptides identified by mass fingerprinting that match calculated molecular weights.

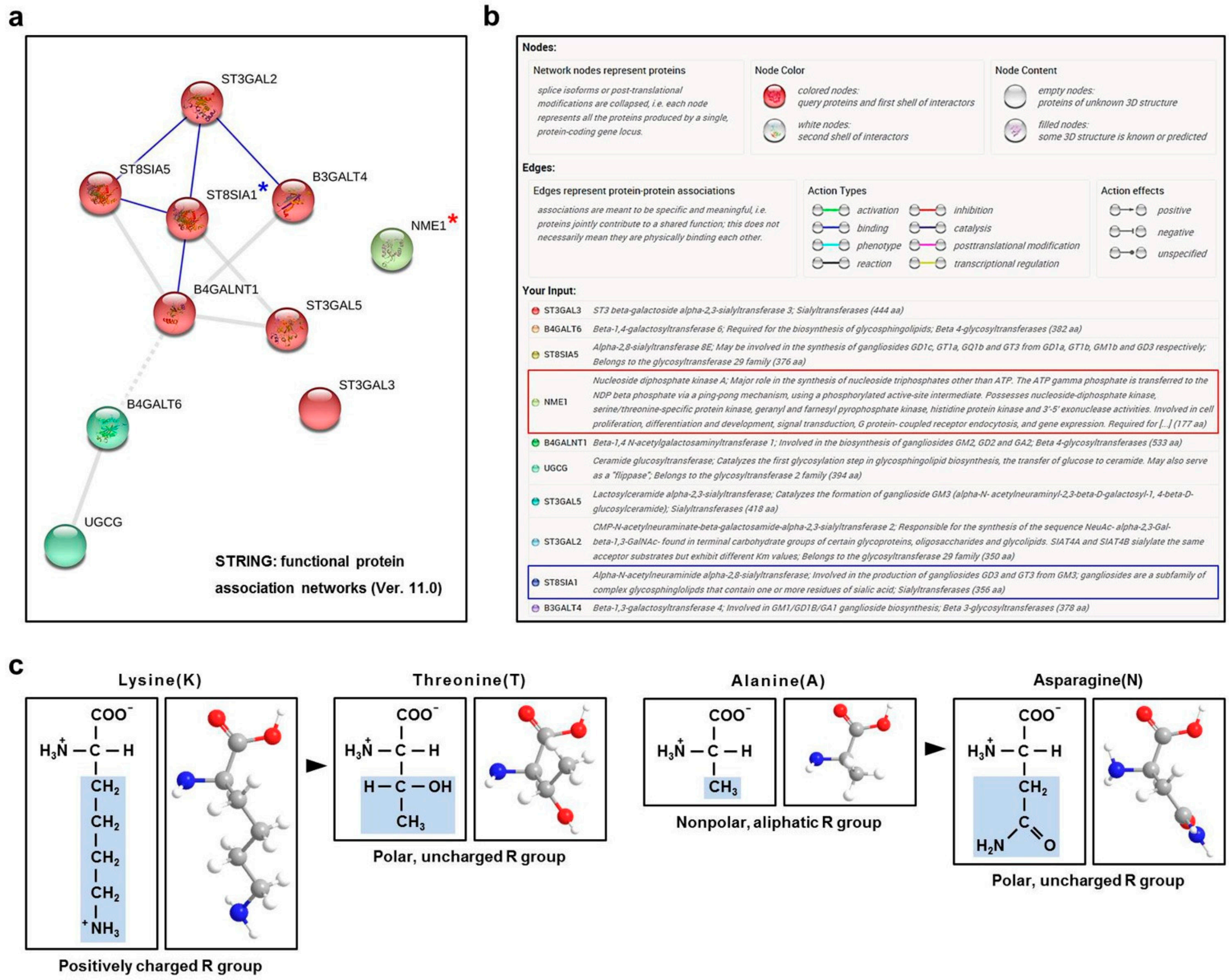

Figure S3. The Protein-protein interaction network for genes and signal analysis legend. **(a)** The protein–protein interaction network for the differentially expressed genes. The lines represent the strength of predicted functional interactions between proteins. The interaction of ST8SIA1 (blue asterisk) and NME1 (red asterisk) proteins was schematized through the STRING database; the interaction of ST8SIA1 and NME1 proteins has not yet been reported. STRING, Search tool for the retrieval of interacting genes/proteins. **(b)** Signal analysis legend. Nodes represent genes; edges represent the corresponding protein–protein interaction pairs. In total, 10 genes were integrated into the network. **(c)** Structural formulas of lysine (K), threonine (T), alanine (A), and asparagine (N).

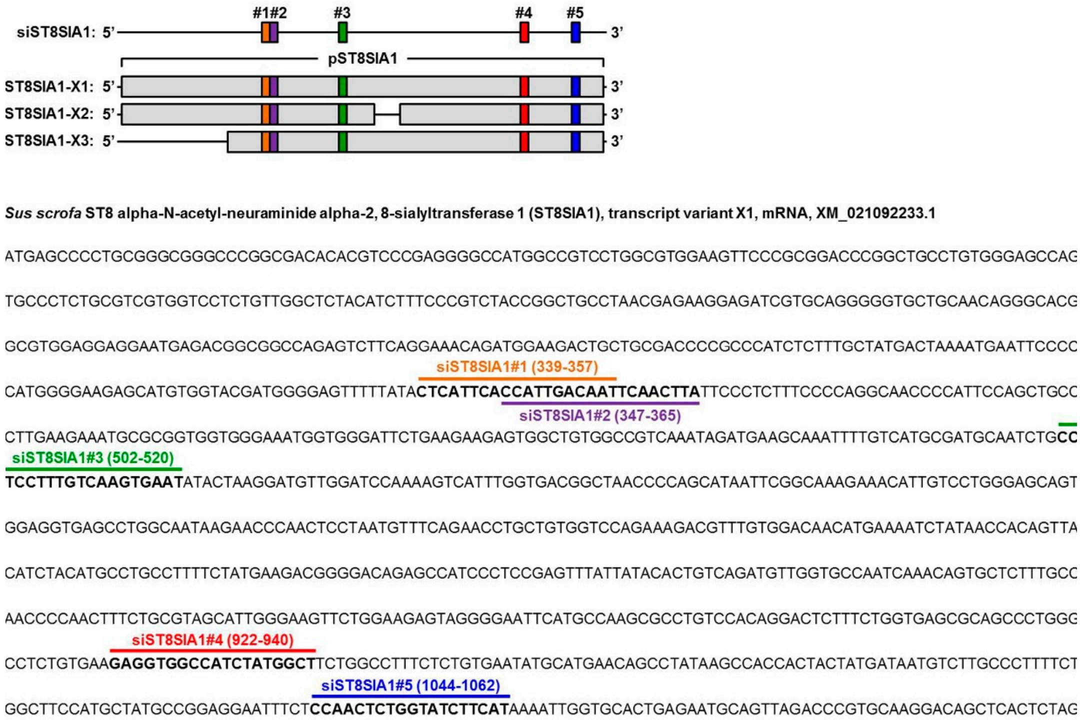

Figure S4. The siRNA information of ST8SIA1. **(a)** The schematic structure of siST8SIA1 and silencing locations on the three ST8SIA1 genes. **(b)** Five siRNA targets were selected in the ST8SIA1 mRNA sequence.

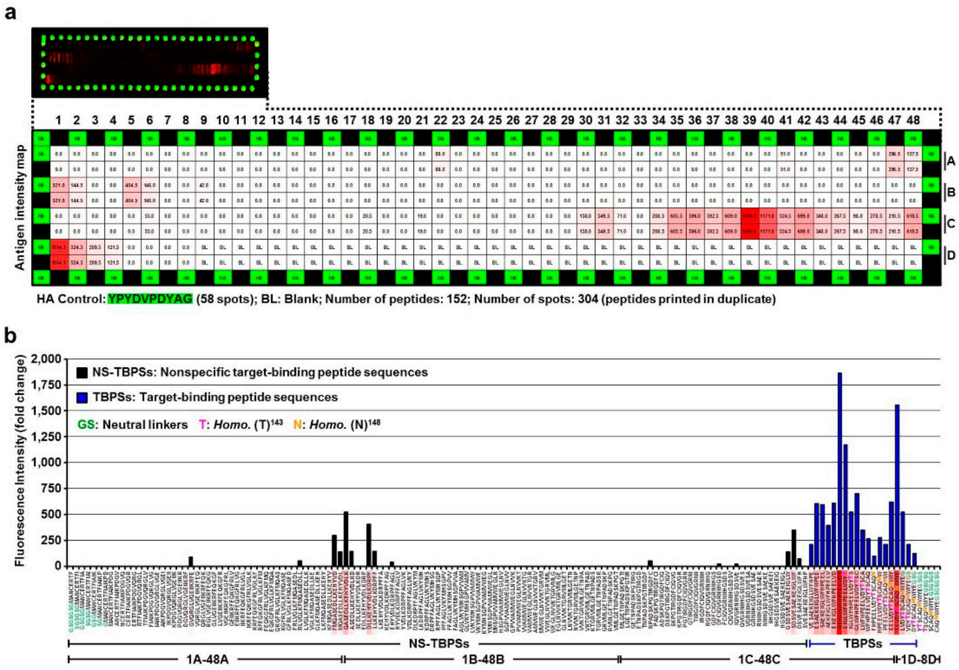

Figure S5. Antigen intensity map and fluorescence intensity of the peptide array signals. **(a)** Antigen intensity map of hNME1 and **(b)** fluorescence intensity of the peptide array signals. Two of Table 143. (T^{143}) and asparagine 148 (N^{148}) in the hNME1 and pNME1 sequence.

International Journal of *Molecular Sciences*

MDPI

Table S1. Primer sequences used to generate templates for RT-PCR.

Gene	Description	Species	Primer (5′→3′)	Reaction condition	Size (bp)
MAP2	Microtubule-associated protein 2	*Sus scrofa*	F: AAG AGA AAG GGG CAA CGC A<br>R: TGG CAC TCT TTT CGC TTG GT	Denaturation: 30 s at 95 °C<br>Annealing: 30 s at 62 °C<br>Extension: 30 s at 72 °C<br>Reaction cycles: 35	375
NF-M	Neurofilament medium	*Sus scrofa*	F: ATA CAC ACC GAC AGC CCT CC<br>R: CCT CTT CGG CTT GGT CTG ATT		390
Nestin	Nestin	*Sus scrofa*	F: GGG TGC AAC GAG AAC TGG AT<br>R: ACA CGG GCT CTA TCA CCT CT		330
GFAP	Glial fibrillary acidic protein	*Sus scrofa*	F: TCG ACC AAC TCA CCG CCA A<br>R: GCT TGG TCA CGT CCA TTT CCA		306
ACTB	β-Actin	*Sus scrofa*	F: CGC AAG TAC TCC GTG TGG AT<br>R: CAG GGC TTA CCT GTA CAC TGA C		727
CD14	Cluster of differentiation molecule 14	*Homo sapiens*	F: GCT GGA CGA TGA AGA TTT CC<br>R: ATT GTC AGA CAG GTC TAG GC	Denaturation: 30 s at 95 °C<br>Annealing: 30 s at 58 °C<br>Extension: 60 s at 72 °C<br>Reaction cycles: 35	535
CD11b	Integrin alpha M	*Homo sapiens*	F: GGT GGC AGT GTG ATG CTG TT<br>R: CCT TGC CTT TCA CCA CCT GA		435
ACTB	β-Actin	*Homo sapiens*	F: CGC AAG TAC TCC GTG TGG AT<br>R: CAG GGC TTA CCT GTA CAC TGA C		687
NME1	Nucleoside diphosphate kinase A	*Homo sapiens*	F: GCC GGA GTT CAA ACC TAA G<br>R: GGA AGG AGG GGA AAT GGA TG	Denaturation: 30 s at 95 °C<br>Annealing: 30 s at 56 °C<br>Extension: 60 s at 72 °C<br>Reaction cycles: 35	543
NME2	Nucleoside diphosphate kinase B	*Homo sapiens*	F: CAC CTT CAT CGC CAT CAA G<br>R: AGA ATG ATC AAT CCT GTT GCC		541
NME1/2	Nucleoside diphosphate kinase A/B	*Homo sapiens*	F: GCC GGA GTT CAA ACC TAA G<br>R: AGA ATG ATC AAT CCT GTT GCC		937

Table S2. Identification of secretomes by MALDI-TOF MS in PMA-induced human MPs.

No.[a]	Gene Name	Accession Number	Description	Score	Expect	M_r[b]	pI[c]
1	MMP9	NP_004985.2	Matrix metalloproteinase-9 preproprotein	198	5e-15	78408	5.69
2	HSPA8	NP_694881.1	Heat shock cognate 71 kDa protein isoform 2	186	7.9e-14	53484	5.62
3	HSPA1A	CAA82315.1	Heat shock 70 kDa protein 1	106	0.0032	69947	5.55
4	PKM	AAH35198.1	Pyruvate kinase, muscle	214	1.3e-16	57942	7.96
5	MMP1	AAV38949.1	Matrix metalloproteinase 1	137	6.3e-09	53928	6.50
6	MMP1	BAD96776.1	Matrix metalloproteinase 1	136	7.9e-09	53948	6.36
7	ENO1	NP_001340275.1	Alpha-enolase isoform 3	201	2.5e-15	47297	6.57
8	CTSD	NP_001900.1	Cathepsin D preproprotein	80	0.0033	44524	8.92
9	MMP1	BAD96755.1	Matrix metalloproteinase 1 preproprotein variant	149	4e-10	53901	6.67
10	MDH2	CAG38785.1	Malate dehydrogenase 2	128	5e-08	35537	8.92
11	PGAM1	AAH62302.1	Phosphoglycerate mutase 1	108	5e-06	28802	6.67
12	TPI1	BAD96358.1	Triosephosphate isomerase 1 variant	150	3.1e-10	26696	6.90
13	GLO1	NP_006699.2	Lactoylglutathione lyase	92	0.0002	20764	5.12
14	PRDX1	EAX06977.1	Peroxiredoxin 1, isoform CRA_b	126	7.9e-08	20715	6.41
15	SOD1	NP_000445.1	Superoxide dismutase	69	0.036	15926	5.70
16	NME1	NP_937818.1	Nucleoside diphosphate kinase A	142	2.1e-09	19641	5.42
17	LYZ	1LZS_A	Lysozyme	111	2.5e-06	14691	9.28

[a]Gel band number; [b]Monoisotopic mass; [c]Isoelectronic point.

Table S3. Information of siRNA sequences for pST8SIA1 gene silencing.

No.	siRNA	Sense (5′→3′)	Antisense (5′→3′)	Position[a]
1	ST8SIA1 #1	CUCAUUCACCAUUGACAAUUU	AUUGUCAAUGGUGAAUGAGUU	339-357
2	ST8SIA1 #2	CCAUUGACAAUUCAACUUAUU	UAAGUUGAAUUGUCAAUGGUU	347-365
3	ST8SIA1 #3	CCUCCUUUGUCAAGUGAAUUU	AUUCACUUGACAAAGGAGGUU	502-520
4	ST8SIA1 #4	GAGGUGGCCAUCUAUGGCUUU	AGCCAUAGAUGGCCACCUCUU	922-940
5	ST8SIA1 #5	CCAACUCUGGUAUCUUCAUUU	AUGAAGAUACCAGAGUUGGUU	1044-1062
6	Scramble	CCUCGUGCCGUUCCAUCAGGUAGUU	CUACCUGAUGGAACGGCACGAGGUU	

All siRNAs were synthesized with 3′-UU overhangs. [a]The position refers to the siRNA sequence position on the target gene.
